# Controversy Surrounding the Function of SpiC Protein in *Salmonella*: An Overview

**DOI:** 10.3389/fmicb.2019.01784

**Published:** 2019-08-07

**Authors:** Yaonan Wang, Yuan Cai, Jian Zhang, Dong Liu, Xiao Gong, Zhiming Pan, Shizhong Geng, Xin’an Jiao

**Affiliations:** ^1^College of Bioscience and Biotechnology and College of Veterinary Medicine, Jiangsu Key Laboratory of Zoonosis, Jiangsu Co-Innovation Center for Prevention and Control of Important Animal Infectious Diseases and Zoonoses, Yangzhou University, Yangzhou, China; ^2^Research and Development Center, State Key Laboratory of Genetically Engineered Veterinary Vaccines, Yebio Bioengineering Co., Ltd of Qingdao, Qingdao, China

**Keywords:** *salmonella*, T3SS2, SpiC protein, virulence factor, pathogenesis

## Abstract

*Salmonella* is an important pathogenic microorganism that can infect humans and animals and has been studied globally as a model microorganism for its pathogenesis. The SpiC protein of T3SS2 is a significant factor that has been studied for almost 20 years, but to date, the function/effect of SpiC in the pathogenesis of *Salmonella* has not been completely understood. There is controversy over the functions of SpiC protein in the literature. Thus, an overview of the literature on SpiC protein is provided here which highlights expression features of SpiC protein and its various functions and effect.

## Introduction

*Salmonella* spp. is an important food-borne zoonotic pathogen, belonging to the family *Enterobacteriaceae*, which infects millions of people and livestock, even leading to human and animal death causing severe economic losses every year (Taylor et al., 2018). It had two species including *Salmonella* Bongori and *Salmonella enterica* and six subspecies in the specie *Salmonella enterica* (Gal-Mora, 2019), over 2,600 serovars have been found (Geng et al., 2016).

Twenty-three *Salmonella* pathogenicity islands (SPIs) have been identified to be involved in *Salmonella* pathogenic mechanism (Fookes et al., 2011; Hayward et al., 2013). Among them, SPI1 and SPI2 are two major SPIs, encoding virulence-related type III secretion system (T3SS), T3SS1 and T3SS2, respectively. Proteins secreted by these two SPIs are involved in pathogenicity at the molecular level and provide a novel insight into *Salmonella* pathogenesis. T3SS2 is not found in *Salmonella* Bongori, but in six subspecies of *Salmonella* enterica. Low pH and nutrient shortage in the *Salmonella*-containing vacuole (SCV) can induce intracellular expression of the T3SS2 (Rappl et al., 2003; Allam et al., 2012). These secreted effectors can modulate the intracellular environment (Deng et al., 2017). During *Salmonella* infection, a variety of virulence factors and effectors are expressed to be translocated across the membrane of the SCV to cytoplasm of infected host cell by the T3SS2 and interact with proteins in host cell to antagonize innate immune defense of host cell, supporting its survival and replication for persistent infection (Schleker et al., 2015).

SpiC was the first SPI2-encoded protein to be identified that could be secreted by the Spi/Ssa T3SS2 system into the macrophage cytoplasm (Ochman et al., 1996; Uchiya et al., 1999). A *spiC*-deletion mutant had significantly reduced virulence in mice. However, the function of the SpiC protein remains unclear and is controversial (Kujat Choy et al., 2004; Figueira and Holden, 2012). It is, therefore, necessary to summarize recent works on the function of SpiC as a reference for future studies. The controversies are presented as follows:

### The Size of SpiC Protein: 133 aa or 127 aa

SpiC, as a virulence factor, is species-specific, comparing with disease pathology between *Salmonella* and *Shigella*. This protein has two isoforms: 133 amino acid (aa) or 127 aa, depending on the translational start site. Presumably, the 127-amino acid ORF is translated form “AGGAG,” the sequence of its putative ribosome-binding site at the 3′ end of the 16S rRNA (Ochman et al., 1996). In the first published paper, the 127 aa isoform was studied (Ochman et al., 1996). As an effector protein, SpiC was suggested to be expressed by SPI2 (Ochman et al., 1996; Hensel et al., 1998; Uchiya et al., 1999) without the conserved N-terminal amino acids, which exists in most of translocated effectors that have been confirmed (Miao and Miller, 2000).

But in many studies, the 133 aa isoform was studied. For example, the level of intramacrophage expression of the *spiC* gene with 402 bp (133 aa) is 52.99-fold greater than that of the ESP (ESP refers to bacterial growth in Lennox broth to OD_600_ = 2.0) (Srikumar et al., 2015).

An adenine is the transcription start site of *spiC*, at 18 basepases upstream of initiation codon (ATG) of the *spiC* gene, which is consistent with the finding of Walthers et al. (Walthers et al., 2007). SpiC protein contains 127 amino acids, with a molecular weight of approximately 14.7 kDa.

### Which One Controls SpiC Expression: T3SS1 or T3SS2?

SpiC is expressed during all stages of infection by T3SS2 and *spiC* gene usually represents T3SS2 in the literature (Bruce et al., 2018); however, SpiC/SsaB is secreted in the early stages of SCV biogenesis (<30 min) by T3SS1 (Steele-Mortimer, 2008).

### Where Do *Salmonella* Express SpiC: Inside or Outside of Infected Cells?

#### Intracellular Expression Inside of Infected Cells

SpiC protein expressed in *Salmonella* might be a regulator of, or chaperone for, the translocon protein SseBC (Freeman et al., 2002); SpiC may control the expression of flagella and fliC since their expression level was lower in *Salmonella* spiC-deleted mutant than that in parent *Salmonella* (Uchiya and Nikai, 2009). SpiC acts as a “gatekeeper” enabling translocon proteins, such as SseBC and SseJ, to be secreted from *Salmonella* (Büttner, 2012). SpiC-2HA, a tagged protein, could only been found in bacteria, not in the cytoplasm and membrane of infected cells (Yu et al., 2002, 2004). *Salmonella* in the SCV do not secrete SpiC into the cytoplasm.

After extracelluar growth of *Salmonella spiC*-deleted mutant and its complementary strain at low pH to induce SPI2-encoded protein secretion. The SpiC protein cannot be detected in *Salmonella* culture media or on the tube inner surface (Shea et al., 1999; Yu et al., 2002). These results are consistent with the research result of Hansen-Wester (Hansen-Wester et al., 2002), a SpiC::M45 tagged protein cannot be detected under similar culture conditions (Yu et al., 2002).

SpiC protein might be a non-effector of the SPI2 T3SS because of failed attempts to show that SpiC was a translocated protein. It might be one kind of SPI2-encoded translocons, but not an effector (Freeman et al., 2002; Yu et al., 2002).

When a host cell is attacked, *Salmonella* Typhimurium expresses a higher level of *spiC mRNA* than *Salmonella* Infantis to survive within macrophages (Braukmann et al., 2015).

#### Extracellular Expression Outside of Infected Cells

In addition to being a member of the regulatory complex, SpiC is secreted by T3SS2 into the eukaryotic cytosol to interfere with cellular behavior (Ramos-Morales, 2012). SpiC can be translocated into the macrophage cytoplasm (Uchiya et al., 1999). It can also act with Hook3 and TassC in the cytosol to interfere with vesicle trafficking to block SCV from fusing with lysosomes and incoming endosomes (Kuhle and Hensel, 2004; Valdez et al., 2009). Thus, *Salmonella* in the SCV can secrete SpiC into the cytosol. *Salmonella*-containing vacuoles normally fuse with lysosomes within 20 min of infection. SpiC may have profound effects on the host cell to block this fusion. SpiC cannot be detected until approximately 1 h after infection. SpiC production may begin while the bacteria are in other cells, but before they enter macrophages, because macrophages rely on vesicle fusion to secrete factors that stimulate and attract other cells of the immune system (Strauss, 1999).

In addition, PspiC (promoter of s*piC*) and PssaG (promoter of *ssaG*) strains were constructed by promoter fusion for RIVET analysis of *spiC* transcription. Typical SPI2 expression features were proved during *in vitro* culture. After PspiC and PssaG strains were orally infected into mouse ileal loops, SpiC protein could also be detected at 15 min post infection (Brown et al., 2005). These results show that SpiC protein can be expressed extracellularly in a normal culture.

Wild-type *Salmonella* harbouring a transcriptional fusion of the *spiC* promoter to a promoter-less *gfp* gene in plasmid pFPV25 showed high levels of *spiC* expression 6 h after infection. This resulted in an 11-fold induction of *intramacrophages spiC* expression when compared with that in tissue culture media (Bijlsma and Groisman, 2005). A *spiC*::lacZ fusion transcription could be detected in *in vitro* culture of *Salmonella* in low-osmolarity N salt medium (Deiwick et al., 1999). Fimbriae with an integrated viral epitope can be induced to expression from the *spiC* promoter in LB containing different concentrations of Mg^2+^ in a cya-crp-pgtE *Salmonella* mutant (Chen and Schifferli, 2003).

*Salmonella* Paratyphi A 45157 and *Salmonella* Typhimurium SL1344 were cultured to the stationary phase *in vitro* at 37 or 42°C under microaerophilic conditions and harvested for total RNA, which was performed by qpCR, mRNA level of *spiC* gene was increased by 30-fold at 42°C compared with that at 37°C (Elhadad et al., 2015).

Organelles such as the SCV, lysosomes (Hashim et al., 2000), phagosomes (Buchmeier and Heffron, 1991), and Golgi body (Shotland et al., 2003) and proteins such as transferrin (Uchiya et al., 1999; Pan et al., 2010), Rab (Buchmeier and Heffron, 1991), HooK3 (Shotland et al., 2003), TassC and NIPSNAP (NSP4) (Lee et al., 2002) interact in the cell. SpiC protein from *Salmonella* can interact with Hook3 protein to affect cellular trafficking (Shotland et al., 2003; Valdez et al., 2009), SpiC protein could enter into the cytoplasm of *Salmonella*-infected cells.

### SpiC Invovle in Virulence: Virulence or Non-virulence Proteins

#### Virulence Protein

The LD_50_ of the *spiC*-inactivated *Salmonella* Pullorum S06004 was 1.2 × 10^9^ CFU, and up to 200-fold greater compared with that of the wild-type S06004 (6.0 × 10^6^ CFU) (Geng et al., 2014). Similar results were obtained for *Salmonella* Gallinarum (Cheng et al., 2016).

The LD_50_ (2.87 × 10^6^ CFU) of a *spiC*-deleted *Salmonella* enteritidis C50041 was 900-fold higher than that of the wild-type strain (3.11 × 10^3^ CFU) when chickens were infected (Zeng et al., 2015). Another research proposed that the *spiC*-deleted mutant of *Salmonella* Typhimurium was of low virulence in intraperitoneally inoculated mice. The LD_50_ of the *spiC-*deleted mutant was over 3.63 × 10^6^ CFU, but the LD50 of wild-type *Salmonella* Typhimurium was below 10 CFU (Uchiya et al., 1999).

Intracellular survival and replication are important virulence determinants (Ibarra and Steele-Mortimer, 2009). The intramacrophage growth of *Salmonella enteritidis* C50041 with a *spiC*-deletion was continuous, while the number of wild-type *Salmonella* began to reduce from 10 to 24 h post infection, but the load of *spiC*-deleted mutants began to increase, which was significantly higher compared with that of the wild-type *Salmonella* in RAW264.7 macrophages at 24 h post infection (Data by our Lab).

Statistical analysis of Grant’s research showed the load of *Salmonella* SPI2 mutants was higher per cell than that of wild-type *Salmonella*. The *ssaV*, *ssaM*, *spiC*, and *sseB* mutants can survive in far fewer infected cells per organ, with higher loads per cell than that of the wild type (Grant et al., 2012). SpiC was necessary for *Salmonella* Typhimurium spread in the spleen (Grant et al., 2012). The intracellular *spiC*-deleted mutant was suggested to change the metabolic pathway for its proliferation using NO (Nitric oxide) as a nitrogen source (Das et al., 2009).

However, Uchiya’s research results showed that the *spiC*-deleted mutant of *Salmonella* Typhimurium could not survive within macrophages (Uchiya et al., 1999). The intramacrophage survival of the *spiC*-deleted mutant of *Salmonella* Typhimurium 14028s was less than 20% compared with 100% of wild-type *Salmonella* Typhimurium 14028s in J774 macrophages at 18 h post infection (Uchiya et al., 1999). In the typhoid model, Buckner et al. found that Δ*spiC* mutant was attenuated for colonization of intestinal and systemic sites. The Δ*spiC* mutant replicated to the same extent as the wild type in epithelial cells but replicated to a poorer extent in macrophages (Buckner et al., 2011).

#### Non-virulence Protein

In an unpublished report by Ajay Singh, the function of the SpiC protein was also analyzed in *Caenorhabditis elegans,* another model of *Salmonella* Typhimurium infection. The conclusions showed that the loss of *spiC* did not change the virulence of *Salmonella* Typhimurium and the lethality of infection in *Caenorhabditis elegans* because of the similar rate of death with the wild-type *Salmonella* group.

### Where SpiC Function/Effect: In *Salmonella* or the Host Cells

#### In *Salmonella*

A *spiC*-deleted mutant of *Salmonella* Typhimurium could not secrete SPI2 proteins again (Yu et al., 2002). After host cells are infected by *Salmonella*, the bacteria can survive in the SCV in the cell and transfer the translocon protein SseBC; the secretion of these effectors is regulated by SpiC protein (Freeman et al., 2002) through the needle of the T3SS to link the SCV membrane. Theoretically, translocon proteins must be expressed and secreted to guide effectors to be translocated. In an *in vitro* assay, SpiC was one component of SsaL/SsaM/SpiC complexes to act as a “gatekeeper” to enable translocon and effector protein secretion at pH 5.0, when wild-type *Salmonella* was exposed to pH 7.2 of host cell cytoplasm by the translocon pore and the canal of the needle at pH 5.0 and dissociation and degradation of SsaM/SpiC/SsaL complexes are brought about (Rappl et al., 2003; Yu et al., 2010). SpiC was therefore deemed to be a switch regulator (Walthers et al., 2007).

The SPI2 effector SseJ and PipB are oversecreted in *in vitro ssaM* and *spiC*-deleted mutants. Immunoblot analysis revealed that at most 5% of the total SpiC protein could be translocated into host cell cytoplasm (Yu et al., 2004). SpiC protein participated in the synthesis of FliC, which could activate SPI2-dependent MAPK pathways in macrophages infected by *Salmonella* (Uchiya and Nikai, 2008).

There were many flagella filaments on the wild-type *Salmonella* surface, while only a few flagella could be observed on the *spiC*-deleted mutant surface. SpiC participated in flagellum assembly by changing flhDC gene expression, which is a primary regulator of flagella synthesis and assembly. The *spiC*-deleted mutant of *Salmonella* Typhimurium is defective in flagella filament formation and shows a severe defect in motility (Uchiya and Nikai, 2009; Valdez et al., 2009). However, our results showed no change in flagella and swim/swarm ability between the *spiC-*deleted mutant and wild-type *Salmonella* enteritidis C50041 (Valdez et al., 2009).

#### In Cells

SpiC can interact with its target proteins in host cells but it does not serve as an independent toxin-like effector (Lee et al., 2002). An intracellular hook protein and the NIPSNAP homolog was confirmed to be a target of SpiC protein (Shotland et al., 2003).

SpiC protein blocks cellular traffic (just as one crucial accident can slow activity throughout a city) and interrupts *Salmonella*-containing vesicle fusion with lysosomes. In general, macrophages rely on vesicle fusion (*Salmonella*-containing vesicles fuse with lysosomes) (Hashim et al., 2000) to secrete factors that stimulate and attract other cells of the immune system. SpiC-mediated blockage of vesicle fusion might also affect macrophage activity in unanticipated ways and hamper immune system functioning (Strauss, 1999). This disruption protects pathogens by antagonizing against cell’s bactericidal contents, such as reactive oxygen and reactive nitrogen species (Kaur and Jain, 2012).

Compared with the *spiC*-deleted *Salmonella*, prostaglandin E2 (PGE2) and prostacyclin (PGI2) were highly expressed and activated the PKA signaling pathway by corresponding receptors and the phosphorylation of ERK1/2 was higher level in J774 macrophages infected by parent *Salmonella* (Uchiya and Nikai, 2004). MAPK pathways in macrophages infected by *Salmonella* could be activated SPI2-dependently, SpiC as major effector regulates the expression of FliC, which is involved in MAPK pathways (Uchiya and Nikai, 2008; Valdez et al., 2009). JAK/STAT signaling pathway could be inhibited by cytokine signaling 3 (SOCS-3), SOCS-3 was highly expressed in J774 macrophages infected by parent *Salmonella* compared with that by *spiC-*deleted mutant. *Salmonella causing spiC*-dependent ERK1/2 activation and then leading to SOCS-3 expression (Uchiya and Nikai, 2005; Valdez et al., 2009). Interleukin-10 (IL-10) mRNA was highly expressed in macrophages infected by parent *Salmonella* compared with that by *spiC*-deleted mutant (Uchiya et al., 2004). Cyclic AMP-dependent PKA showed higher activity in macrophages infected by parent *Salmonella* compared with that by *spiC*-deleted mutant, but there were no obvious difference of the levels of IL-1β, IL-6, and TNFα (Uchiya and Nikai, 2004).

The assembly of F-actin protein was regulated by SpiC in intracellular *Salmonella* Typhimurium (Holden, 2002; Yu et al., 2002). A *spiC*-deleted mutant cannot form SCV filaments, suggesting that another SPI2 effector SifA may not translocate in this *spiC*-deleted mutant (Lee et al., 2002; Valdez et al., 2009). *spiC*-deleted ST240 can not produce completely generate lgp (lysosomal membrane glycoprotein)-tubules within HeLa cells (Guy et al., 2000). SIF formation is controlled by *SpiC*, SseF, and SseG (Kuhle and Hensel, 2004). SIF formation and translocons secretion in the *spiC* complementary *Salmonella* based on plasmid-expression was restored (Yu et al., 2004).

#### Other Functions/Effect

SPI2 mutant strains were attenuated *in vivo*, showing reduced tissue colonization and enhanced T-cell activation, which confers protection against a challenge with wild-type virulent *Salmonella* (Tobar et al., 2006). SpiC was also suggested to inhibit dendritic cell movement and antigen delivery (McLaughlin et al., 2014), thus inhibiting Salmonella from escaping the infected cell to spread throughout the body and mediating Salmonella to antagonize (resist) host cell defenses, including respiratory burst (NO, H_2_O_2_, NADPH), the inflammatory response, and cellular autophagy (Vazquez-Torres et al., 2000; McCollister et al., 2005; Bourret et al., 2009; Das et al., 2009). The *spiC-deleted* mutant complementated with a plasmid containing *spiC* allele restored the P-body (dynamic aggregates of RNA and proteins) disassembly phenotype (Eulalio et al., 2011).

In our laboratory, the *spiC* mutant of *Salmonella* Pullorum which was highly attenuated was found to persist in spleen and liver for less than 10 days and induced high levels of circulating antibody and protective immunity against oral challenge in young broiler chickens. It showed that the *spiC* mutant is a potential new vaccine candidate for use with chickens against this disease (Geng et al., 2014). Then 1009Δ*spiC*Δ*crp*, a *spiC* and *crp* deletion mutant of *Salmonella* Gallinarum, was evaluated in chickens. 1009Δ*spiC*Δ*crp* bacteria colonized and persisted in the liver and spleen of vaccinated chickens for >14 days, and significant specific humoral and cellular immune responses were induced. Efficient protection was observed after vaccinated chickens were challenged with *Salmonella* Gallinarum SG9 at 21 days post-immunization. These results demonstrate that 1009Δ*spiC*Δ*crp* can be used as a live attenuated vaccine (Cheng et al., 2016). In addition, *Salmonella* Pullorum Δ*spiC* Δ*waaL* was found to facilitate the differentiation between infected and vaccinated chickens. What is more, the vaccine candidate showed adequate safety being avirulent in the host chicks. Single intramuscular immunization of day-old broiler chicks with the mutant confers ideal protection against lethal wild-type challenge by significantly stimulating both humoral and cellular immune responses as well as reducing the colonization of the challenge strain. The mutant strain generated cross-protection against challenge with the wild-type *Salmonella* Gallinarum. These results suggest that the double-mutant strain may be a safe, effective, and cross-protective vaccine against *Salmonella* infection in chicks while conforming to the requirements of the DIVA program (Guo et al., 2017).

However, the previous studies were all related to vaccine research and development. The underlying mechanisms remain unclear, which is needed to be studied in the future.

## Author Contributions

YW, YC, and JZ collected all references. YW and SG drafted the manuscript. DL, XG, ZP, and XJ revised and finalized the manuscript. All authors read and approved the final version of the manuscript.

### Conflict of Interest Statement

The authors declare that the research was conducted in the absence of any commercial or financial relationships that could be construed as a potential conflict of interest.
